# Polycyclic Aromatic Hydrocarbon Residues in Serum Samples of Autopsied Individuals from Tennessee

**DOI:** 10.3390/ijerph120100322

**Published:** 2014-12-25

**Authors:** Aramandla Ramesh, Anil Kumar, Mounika P. Aramandla, Alfred M. Nyanda

**Affiliations:** 1 Department of Biochemistry & Cancer Biology, Meharry Medical College, 1005 D.B. Todd Blvd., Nashville, TN 37208, USA; 2 Department of Neuroscience & Pharmacology, Meharry Medical College, 1005 D.B. Todd Blvd., Nashville, TN 37208, USA; E-Mail: akumar@mmc.edu; 3 M.L. King Magnet High School, 613 17th Ave North, Nashville, TN 37203, USA; E-Mail: mounikaaramandla26@gmail.com; 4 Department of Professional & Medical Education, Meharry Medical College, 1005 D.B. Todd Blvd., Nashville, TN 37208, USA

**Keywords:** polycyclic aromatic hydrocarbons, benzo(a)pyrene, body burden, autopsy, serum, postmortem

## Abstract

This study reports the concentrations of Polycyclic Aromatic Hydrocarbons (PAHs) in human blood sera samples (*n* = 650) obtained at autopsy from individuals who died of drug abuse, alcohol toxicity, homicide, suicide and other unknown causes. The analyzed samples from decedents revealed the presence of PAHs of which B(a)P was the most predominant one, followed by benzo(b)fluoranthene and benzo(k)fluoranthene. The other PAHs detected sporadically and measured were benzo(g,h,i)perylene, acenaphthene, anthracene, phenanthrene, and fluoranthene The mean concentrations of PAHs were greater in the twenties to fifties age groups compared to others. The PAH residue levels detected were high in African Americans compared to Caucasians, Asians, and Hispanics. It appears that environmental exposure, dietary intake and in some cases occupational exposure may have contributed to the PAH body burden. While the PAH residue concentrations measured fall within the range of those reported for healthy adults elsewhere, in isolated cases, the concentrations detected were high, calling the need for a reduction in PAH emissions and human biomonitoring studies for purposes of risk assessment.

## 1. Introduction

One of the legacies of the Industrial Revolution is a better quality of life on one the hand and declining environmental quality on the other. The decline in environmental quality is due to the release of a multitude of chemicals from activities associated with everyday life such as transportation, material production, waste disposal, *etc.* A group of chemicals which have been associated with urban sprawl are polycyclic aromatic hydrocarbons (PAHs). Substantial intake of these toxicants in humans occurs through exposure routes such as eating of PAH-contaminated food and drinking water and inhaling gaseous PAHs. Inhalation of particle-borne or gaseous PAHs occur through tobacco smoke, automobile exhausts, and combustion processes, which include burning of coal, oil, and biomass [1,2]. Several occupational categories such as people who handle jet fuel, coke, aluminum industries, and work as fire fighters are exposed to PAHs. Detailed sources of PAH exposures have been listed in Ramesh *et al.* [3,4].

While environmental monitoring will indicate the levels of PAHs in the immediate environment and likelihood of exposure, biological monitoring will be helpful as it integrates exposure from all sources and serves as a good estimate of risk from an individual PAH toxicant or mixtures. The matrices for biological monitoring of PAHs are blood, breast milk and urine [5,6]. However, collection of samples from live subjects entails use of techniques, which may cause some discomfort, and the logistics associated with study approvals, and sample collection for targeted chemicals (PAHs). In this regard, utilization of autopsy samples are a feasible and inexpensive alternative so long as the samples are from subpopulations, which are geographically close and not much time is elapsed between death and sample collection (*post mortem* interval). The purpose of our investigation was to determine the background residue levels of PAHs in humans using autopsy samples as a study matrix. Hence serum samples from autopsied individuals were used in the present study as PAH concentrations in blood could be highly correlated to exposure, absorption and retention, thereby serving as an indicator of exposure to PAHs in some counties in the state of Tennessee.

## 2. Methods

During the years 2001–2003 a total of 650 autopsy samples from 33 counties (Benton, Bledsoe, Cannon, Cheatham, Dekalb, Dickson, Dyer, Franklin, Gibson, Giles, Grundy, Hamilton, Hardeman, Hardin, Henderson, Henry, Houston, Lauderdale, Lawrence, Madison, Marion, Maury, McNairy, Obion, Overton, Putnam, Rhea, Robertson, Rutherford, Shelby, Sumner, Weakley and White) in the State of Tennessee (USA) were referred to our clinical reference laboratory from the respective county medical examiner’s office that oversees and ascertains the causes of death in these counties within its jurisdiction. Our laboratory, which was under accreditation by the state of Tennessee, State of New York and College of American Pathologists (CAP) during the above-mentioned period, was asked to run a full screen for toxicology. The samples were obtained from decedents where the causes of death were including but not limited to categories such as prescription drug abuse, drug overdose, alcohol intoxication, suicide, homicide, arson, vehicular accidents and health maladies ranging from cardiovascular to geriatric issues, and sudden infant death syndrome.

Peripheral blood (femoral or subclavian) samples were harvested from the body during the autopsy by the medical examiner or his designee. The samples were preserved in 10 mg/mL sodium fluoride (preservative) and 3 mg/mL potassium oxalate (anticoagulant), individually bagged in leak-proof containers and tamper-proof packages and were shipped to our laboratory for analyses. Samples were shipped after retrieval at the earliest by the medical examiner’s office to prevent loss of analytes by degradation processes.

To respect individual’s privacy and avoid reporting bias, samples were de-identified except for age, gender, and race. Samples were re-coded by the laboratory director prior to chemical analyses to make the analyst(s) blind to the age, gender, race, and cause of death. After the samples were analyzed, data was scrutinized, and the reports were submitted by the laboratory director to the medical examiner’s office. The guidelines for “chain of custody” set forth for reference labs by the accreditation bodies were duly adhered to during the entire process of sample receiving, chemical analyses, and data reporting. This study conducted with data obtained from postmortem samples was approved by the Institutional Review Board at Meharry Medical College.

### 2.1. Sample Processing

Two milliliters of blood sample was mechanically pipetted into a 16 × 100 mm prelabeled disposable borosilicate tube. Fifty microliters of working internal standard was added to each tube (concentration 250 ng/mL). Then 3 mL of 0.25 M phosphate buffer (pH 9.1) without activator was added to each tube. The contents were centrifuged at 2,500 RPM for 3 min.

### 2.2. Extraction

Samples were extracted using solid phase extraction (SPE) method. The SPE columns (Sigma, St. Louis, MO, USA) were washed with 2 mL methanol and 3 mL 0.25 M phosphate buffer with column activator. The blood samples were poured into the appropriately labeled columns. The samples were eluted at a flow rate of 1.0 mL/min. The columns were washed by passing 5 mL 0.0625 M phosphate buffer, pH 9.1. The extracts were eluted by pipetting 0.5 mL acetonitrile/*n*-butyl chloride elution solvent (55/45) into each tube and the eluates were collected in 10 mL screw capped centrifuge tubes. The solvents were evaporated under a gentle stream of N_2_. Each of the dried residues was dissolved in 50 µL of ethyl acetate and the contents were transferred into gas chromatograph/mass spectrometer vials (Agilent, Wilmington, DE, USA) with glass inserts and screw tops and arranged in the auto sampler turret.

### 2.3. Internal Standards and Calibration

Since a great majority of autopsy samples obtained were from suspected drug abuse, overdose and drug-addiction related offence cases, the samples were screened for prohibited drugs and their metabolites at the request of the medical examiner. Benzoylecgonine (the primary metabolite of cocaine) was used as both a negative (0 ng/mL benzoylecgonine) and positive control (benzoylecgonine at approximately 25% above the cut off) as outlined by National Institute of Drug Abuse. The standard solutions of 100, 150 and 500 ng/mL benzoylecgonine were individually prepared from the 1000 µg/mL stock and used to quantify the recovery. Trimipramine (a tricyclic antidepressant compound) at a concentration of 1000 ng/mL was used as the internal standard, which was prepared from a stock concentration of 1000 µg/mL.

A three-point calibration curve was constructed using the 100, 150 and 500 ng/mL standards. A linear regression calibration file was constructed. If the quantitative data of quality control samples were within ±20% of target value, the accuracy of the calibration curve was deemed acceptable. The limits of detection (LOD: lowest dilution of a positive specimen for which the ion ratios are ±20% of the cutoff calibrator) and limit of quantitation (LOQ: lowest concentration for which quantitation and ion ratios are ±20% of the target values) were set as part of our laboratory’s validation assay protocol. The lower and upper limits of detection for this procedure were 20 ng/mL and 5000 ng/mL benzoylecgonine, respectively. Samples which yielded higher values were diluted and subjected to repeat analysis.

### 2.4. Instrumentation and Chromatographic Conditions

Samples were analyzed by GC-MS using an Agilent Model 5890 gas chromatograph (Agilent Technologies, Delaware, MD, USA) coupled with an Agilent Model 5970/5971 mass selective detector. Separation was carried out in a GC column HP-1 or HP-5 (Agilent; 12 m × 0.2 mm × 0.33 µm film thickness) using a temperature programmed GC for elution of analytes. The column temperature was set initially at 145 °C and held for 4 min. This was ramped to 275 °C at 10 °C/min. and held for 20 min. The total run time was 34 min. The target ions monitored were 240, 341, and 346 *m*/*z* for benzoylecgonine and 73, 207, 295, and 533 *m*/*z* for trimipramine, respectively. Retention times and measured ion percentages were used for qualitative reporting decisions while peak areas were used for quantitation purposes.

## 3. Results and Discussion

Even though more than 100 different PAH compounds are known to occur in environment and biological samples, the U.S. EPA has classified 16 of the PAHs as priority-pollutants based on information available on frequency of occurrence at hazardous waste sites, other environmental media, toxicity, and potential for human exposure [7]. Out of the EPA 16 PAHs, the PAHs detected in autopsy samples in the present study were benzo(a)pyrene [B(a)P], benzo(b)fluoranthene [B(b)f], benzo(k)fluoranthene [B(k)f], benzo(g,h,i)perylene, acenaphthene, anthracene, phenanthrene, and fluoranthene. Among these, benzo(a)pyrene, benzo(b)fluoranthene, benzo(c)fluoranthene were the most frequently detected ones. This observation could be due to limitations of the analytical method we used, which was meant for detecting and measuring drugs of abuse. Had we used a PAH-specific analytical method, the results, if not the trend could have been different.

**Figure 1:**
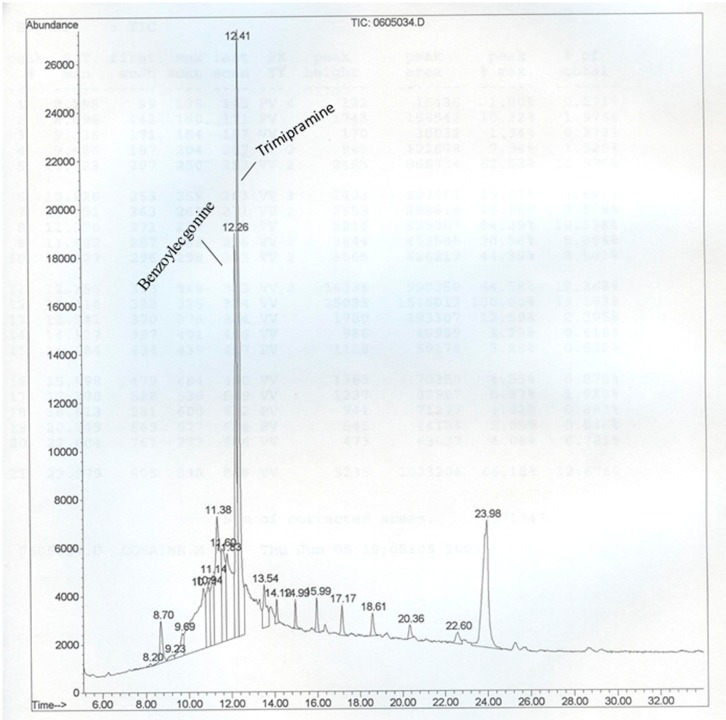
Chromatogram showing peaks of benzoylecgonine (metabolite standard for cocaine) and trimipramine (a tricyclic antidepressant used as internal standard). Since PAHs were detected during screening of autopsy samples for drug abuse, this chromatogram is shown to present the retention time for the cocaine metabolite.

**Figure 2:**
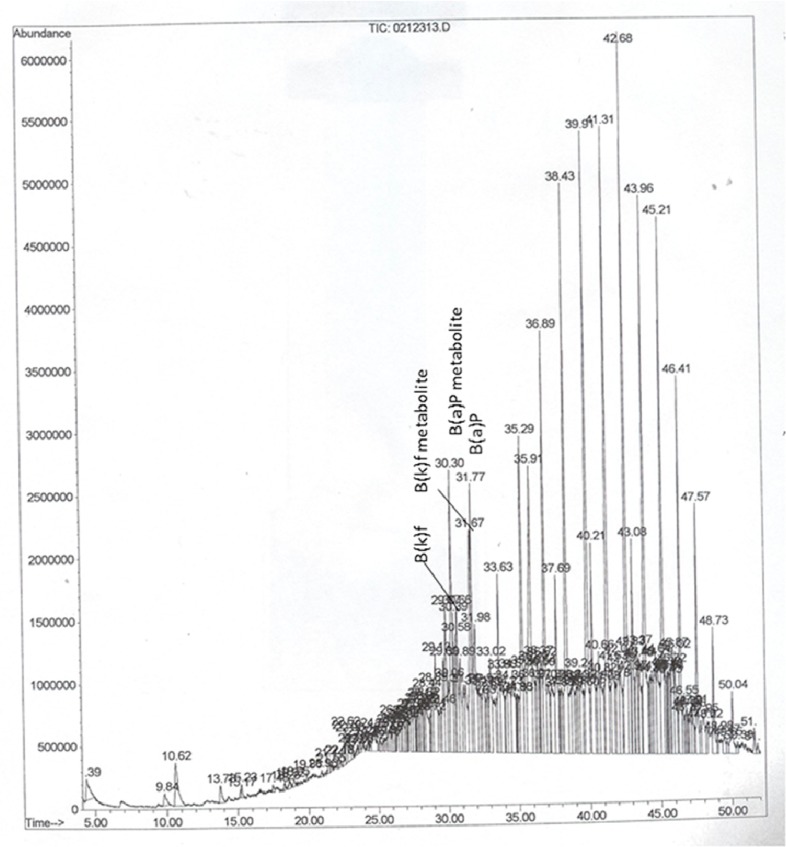
Representative GC-MS chromatogram of PAHs in serum samples of a Caucasian male. The abbreviations are; B(a)P-benzo(a)pyrene, and B(k)f-benzo(k)fluoranthene.

**Figure 3:**
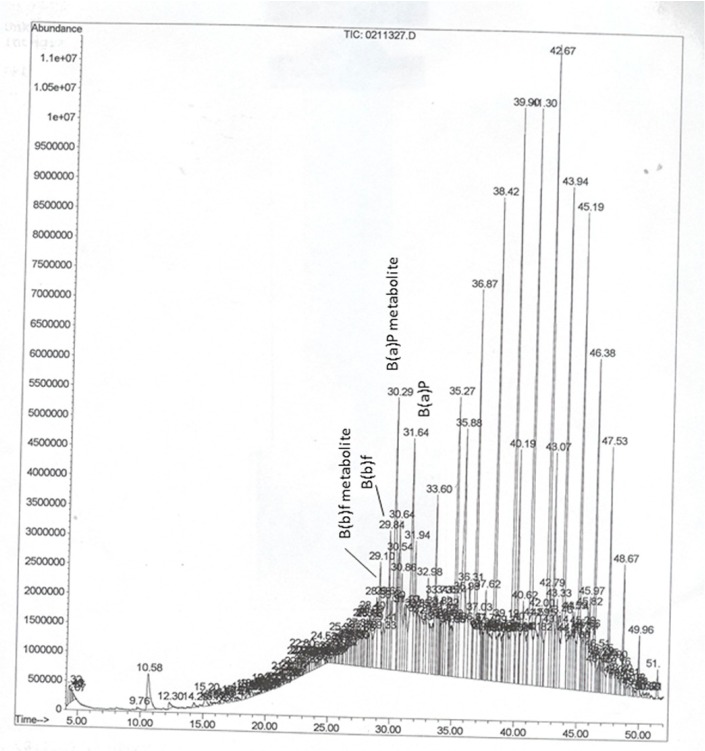
Representative GC-MS chromatogram of PAHs in serum samples of an African American male. The abbreviations are; B(a)P-benzo(a)pyrene, and B(b)f-benzo(b)fluoranthene.

Given that B(a)P and/or other PAHs undergo biotransformation, producing an array of metabolites, questions might arise whether the B(a)P peaks identified from the mass spectrometer include the parent compound as well as the metabolites. In several instances, we have come across multiple peaks that were identified as B(a)P by the mass spectral library. The mass spectral data for B(a)P shows a base peak [8]. The other peaks represent metabolites of B(a)P with different retention times [9] that were resolved by the mass spectrometer. Therefore, for data reporting purposes, the concentrations of all B(a)P derivatives detected and measured during a single run were pooled together to report as the sum total of B(a)P concentrations measured for each sample. Representative chromatograms showing the analytes are presented in Figure 1, Figure 2 and Figure 3. The chromatograms showed a B(a)P peak with a retention time of 31.65 min. The putative B(a)P metabolites showed peaks with retention times of 30.16, 30.29, and 30.30 min. The B(b)f parent compound showed a peak with retention time of 29.84 min, while one of its metabolites showed a peak at 29.11 min. Similarly, the B(k)f parent compound showed a peak with retention time of 31.98 min, while one of its metabolites showed a peak at 31.67 min. Since the objective of our study was not detecting and quantifying individual PAH metabolites, no metabolite standards were used to identify the key metabolites of PAHs. Additionally, since the samples are from autopsy cases whether metabolite stability may have been compromised is not known.

Out of 650 postmortem sera samples received for a drug screen, PAHs were detected in 305 samples. Most samples that were positive for PAHs were from children, teens, and adults in their sixties. Very few samples from septuagenarian and octogenarian decedents showed PAHs. The total PAH (sum of benzo(a)pyrene, benzo(b)fluoranthene, benzo(k)fluoranthene, benzo(g,h,i)perylene, acenaphthene, anthracene, phenanthrene, and fluoranthene) concentrations in serum of autopsy samples exhibited wide variations. The total PAH concentrations (non-lipid adjusted and age stratified; individual data not shown here) ranged from 0.3 to 12 ng/mL, 11 to 16 ng/mL, 0.8 to 19 ng/mL, and 1.1 to 16 ng/mL in in African American men (*n* = 96), African American boys (*n* = 3), African American women (*n* = 56) and African American girls (*n* = 5) respectively. On the other hand, the total PAH concentrations ranged from 0.1 to 12 ng/mL, 4 to 7.6 ng/mL, 0.2 to 13 ng/mL, and 0.64 to 9.7 ng/mL in Caucasian men (*n* = 69), Caucasian boys (*n* = 2), Caucasian women (*n* = 44), and Caucasian girls (*n* = 7) respectively. Compared to the above two racial groups, the number of Hispanic and Asian samples received were low (six Hispanics and two Asians), and when detected the ΣPAH concentrations ranged from 1.5 to 7.5 ng/mL in Hispanic men and 9.1 ng/mL in Asian woman. No samples were received for Hispanic women or Asian men. A single sample of Asian male child showed total PAH concentration of 9.1 ng/mL.

Owing to the limited number of samples in some age groups, in this paper data was not reported on an individual specimen basis in these groups; instead, the concentrations were grouped into children & teens, and adults in their twenties & thirties, forties & fifties, sixties and above categories. The total PAH concentrations from different age groups are shown in Table 1.

**Table 1:** Concentrations (ng/mL) of ΣPAH residues in serum samples from autopsy cases.

Racial Groups	Children & Teens	Twenties & Thirties	Forties & Fifties Male	Sixties & above Female
Caucasian male	5.2 ± 1.5 (3)	4.5 ± 0.76 (25)	4.4 ± 0.55 (39)	3.4 ± 0.96 (14)
Caucasian female	5.2 ± 1.2 (9)	4.4 ± 1.3 (14)	6.2 ± 2.0 (23)	3.9 ± 1.7 (5)
African American male	6.8 ± 2.1 (9)	4.5 ± 0.39 (54)	5.2 ± 0.98 (28)	5.0 ± 1.3 (8)
African American female	5.3 ± 2.5 (7)	6.1 ± 0.87 (39)	9.0 ± 1.84 (13)	6.8 (2)
Hispanic male	ND	3.4 ± 0.78 (5)	7.5 (1)	ND
Hispanic female	ND	ND	ND	ND
Asian male	12.8 (1)	9.1 (1)	ND	ND
Asian female	ND	ND	ND	ND

Values were presented as mean ± standard error. Values in parentheses indicate number of samples that were positive for PAHs. ND denote none of the. PAHs were detected.

The variations in PAH residue levels could be due to inter-individual differences in dietary habits [10], exposure to mainstream or side stream cigarette smoke [11], uptake and biotransformation of the PAHs [12] and other preexisting pathophysiological conditions [13]. Use of autopsy samples for estimating the body burden of PAHs rests on the premise that the PAH concentrations found at postmortem are a measure of the amount of PAHs present at the time of death. Thus, the PAH concentrations in decedents are presumed to reflect either an individual or cumulative exposures to the above-mentioned PAH sources. This extrapolation may have some limitations as postmortem redistribution of drug concentrations in blood is a possibility [14,15]. To minimize this confounder, we have selected data from samples that were taken out from decedents soon after their deaths, but not from exhumed bodies and in situations where the bodies may have been retrieved several hours after the deaths were reported.

Drawing comparisons between our study and those of the studies that involve live subject populations are fraught with difficulties since, to do a meaningful comparison, one has to choose PAH-unexposed study populations that are representative of rural areas or urban areas far from industrial sources. The sample size is another hindrance. It is ideal to have a larger sample size (300–700 subjects) in each category (race, age, gender *etc.*) for estimation of 95% confidence intervals and for obtaining normalized reference levels for skewed distributions [16,17]. To the possible extent, we have used basic statistics to analyze the data. Detailed statistical analyses of the data require robust and systematic data collection as practiced in biomonitoring and epidemiological studies [16]. Such an undertaking in forensic toxicology settings is difficult where the mortalities are unexpected happenings and the cost and time constraints restrict the number of autopsies performed, the type of samples provided to the lab and the tests that were asked for by the medical examiner/coroner.

As regards the composition of PAH residues, B(a)P, B(b)f and B(k)f were measured frequently. The percent composition of PAH residues were presented in Table 2.

**Table 2:** Percent composition of PAH residues in serum samples from autopsy cases.

Racial Groups	Children & Teens			Twenties & Thirties			Forties & Fifties			Sixties & Above		
	B(a)P	B(b)f	Others & B(k)f	B(a)P	B(b)f	Others & B(k)f	B(a)P	B(b)f	Others & B(k)f	B(a)P	B(b)f	Others & B(k)f
Caucasian male	60	40	0	60	35	5	60	35	5	60	35	5
Caucasian female	50	45	5	50	45	5	50	45	5	50	50	0
African American male	60	40	0	60	40	0	60	40	0	60	40	0
African American female	50	50	0	50	47	3	50	45	5	50	45	5
Hispanic male	ND	ND	ND	55	45	0	55	45	0	ND	ND	ND
Hispanic female	ND	ND	ND	ND	ND	ND	ND	ND	ND	ND	ND	ND
Asian male	50	47	3	50	45	5	ND	ND	ND	ND	ND	ND
Asian female	ND	ND	ND	ND	ND	ND	ND	ND	ND	ND	ND	ND

Values were presented as percent composition of individual PAH compound(s) in the sum total of PAHs measured. The abbreviations are; B(a)P-benzo(a)pyrene, B(b)f-benzo(b)fluoranthene, B(k)f-benzo(k)fluoranthene, “others” indicate a mixture of benzo(g.h.i)perylene, acenaphthene, anthracene, and fluoranthene. ND denote none of the PAHs were detected.

Our results are in agreement with those of Seto *et al.* [18] and Beach *et al.* [19] who also detected these PAHs from autopsy and patient tissue samples respectively. While B(a)P is a ubiquitous PAH compound, B(b)f and B(k)f are less prevalent compared to B(a)P, but persist in environmental media [20] and biological samples [21]. These PAHs were a part of the eight nonvolatile PAHs, which registered elevated concentrations in indoor & outdoor air in urban homes in New York and in Poland [22]. Epidemiological studies of traffic police officers in Italy showed predominance of the above-mentioned three PAHs in white blood cells [23]. A survey of blood plasma collected from healthy residents in Hong Kong also revealed the presence of above-mentioned toxicants [24]. Additionally, a correlation between atmospheric levels of these PAHs and urinary nicotine metabolites in smokers have been found in a national survey of general population in the United Kingdom [5].

Samples from African Americans (AA) registered greater levels of PAHs, compared to Caucasians, which could be attributed to polymorphisms in PAH metabolizing enzymes resulting in an increased likelihood of health risk in AA due to PAH exposures from dietary, smoking habits and environment [25,26,27,28]. An interesting finding in this study is that the PAH residue concentrations in children and teens were comparable to the adults indicating that they may have been exposed to PAHs through polluted air and diet in as much as the adults. Repeat analyses of air and urine samples from children residing in New York City revealed substantial indoor exposure to PAHs during heating season [21]. Transfer of PAHs from mothers to fetuses through placenta and to infants through breast milk [29] is another possibility. The PAH residue levels in children raises concern because these toxicants were known to affect cognitive development in young children [30].

To our knowledge, there were no prior reports of PAH residue concentrations in serum of autopsy cases to compare our data with those of others. Nonetheless, the PAH residue concentrations measured in the present study (0.3–19 ng/mL) are well within the range reported in the literature (0.15–5.45 ng/mL [31]; 3.0–231 pg/mL [32]; 0.37–260 ng/mL [33]) for normal population. However, in isolated cases, the concentrations recorded in the present study registered high levels, which could be due to a combination of exposures from diet, and the quality of decedents living environment. Additionally, most of the autopsy samples analyzed were from drug abusers, many of whom may also have been exposed to tobacco smoke in addition to smoke from narcotics. It has already been reported that in some Tennessee soils the total PAH concentrations measured exceeded the EPA preliminary remediation goals [34]. Furthermore, exposure to PAHs emanated from the superfund sites [35] in Tennessee cannot be ruled out. A study conducted by the U.S. Geological Survey revealed that PAHs released from abandoned oil and gas wells in the Big South Fork National River and Recreation Area in Tennessee [36] and PAH contamination from coal mining or natural gas extraction activities in the upper Tennessee River basin and eastern Cumberland River basin [37] may affect the water quality, human activities and aquatic animal life. Episodic release of PAHs from events such as coal ash spills in Kingston, Tennessee [38] also adversely affected environmental and human health. Aside from environmental exposures, life style-associated factors may also have a bearing on PAH-induced illnesses in some Tennessee residents. The Tennessee colonoscopy-based case control studies reported that cigarette smoking [39], and consumption of well-done red meat [40], which are rich sources of PAHs showed a positive association with colorectal cancer risk.

## 4. Conclusions

In conclusion, findings from our study calls the need for (i) raising the awareness of both manufacturers and consumers to control PAHs emissions into the environment; (ii) monitoring the levels of these toxicants in the environment, foodstuffs, and humans; and (iii) enforcing the safe-limits by regulatory agencies for human exposure to PAHs so that the health risk could be minimized.

## References

[1] WHO (2010). IARC Monographs on the evaluation of carcinogenic risks to humans. Some Non-Heterocyclic Polycyclic Aromatic Hydrocarbons and Some Related Exposures.

[2] Choi H., Spengler J. (2014). Source attribution of personal exposure to airborne polycyclic aromatic hydrocarbon mixture using concurrent personal, indoor, and outdoor measurements. Environ. Int..

[3] Ramesh A., Walker S.A., Hood D.B., Guillen M.D., Schneider H., Weyand E.H. (2004). Bioavailability and risk assessment of orally ingested polycyclic aromatic hydrocarbons. Int. J. Toxicol..

[4] Ramesh A., Archibong A.E., Hood D.B., Guo Z., Loganathan B.G., Loganathan B.G., Lam P.K.-S. (2011). Global environmental distribution and human health effects of polycyclic aromatic hydrocarbons. Global Contamination Trends of Persistent Organic Chemicals.

[5] Aquilina N.J., Delgado-Saborit J.M., Meddings C., Baker S., Harrison R.M., Jacob P., Wilson M., Yu L., Duan M., Benowitz N.L. (2010). Environmental and biological monitoring of exposures to PAHs and ETS in the general population. Environ. Int..

[6] Wilhelm M., Eberwein G., Hölzer J., Gladtke D., Angerer J., Marczynski B., Behrendt H., Ring J., Sugiri D., Ranft U. (2007). Influence of industrial sources on children’s health—Hot spot studies in North Rhine Westphalia, Germany. Int. J. Hyg. Environ. Health.

[7] ATSDR (1995). Toxicological Profile for Polycyclic Aromatic Hydrocarbons (PAHs).

[8] Takahashi G., Kinoshita K., Hashimoto K., Yasuhira K. (1979). Identification of benzo(a)pyrene metabolites by gas chromatograph-mass spectrometer. Cancer Res..

[9] Lee W., Shin H.S., Hong J.E., Pyo H., Kim Y. (2003). Studies on the analysis of benzo(a)pyrene and its metabolites in biological samples by using high performance liquid chromatography-fluorescence detection and gas chromatography/mass spectrometry. Bull. Korean Chem. Soc..

[10] Duarte-Salles T., Mendez M.A., Pessoa V., Guxens M., Aguilera I., Kogevinas M., Sunyer J. (2010). Smoking during pregnancy is associated with higher dietary intake of polycyclic aromatic hydrocarbons and poor diet quality. Public Health Nutr..

[11] Castaño-Vinyals G., D’Errico A., Malats N., Kogevinas M. (2004). Biomarkers of exposure to polycyclic aromatic hydrocarbons from environmental air pollution. Occup. Environ. Med..

[12] Zhong Y., Wang J., Carmella S.G., Hochalter J.B., Rauch D., Oliver A., Jensen J., Hatsukami D.K., Upadhyaya P., Zimmerman C. (2011). Metabolism of [D10] phenanthrene to tetraols in smokers for potential lung cancer susceptibility assessment: Comparison of oral and inhalation routes of administration. J. Pharmacol. Exp. Ther..

[13] Gandhi A., Moorthy B., Ghose R. (2012). Drug disposition in pathophysiological conditions. Curr. Drug Metab..

[14] Cook D.S., Braithwaite R.A., Hale K.A. (2000). Estimating antemortem drug concentrations from postmortem blood samples: The influence of postmortem redistribution. J. Clin. Pathol..

[15] Richardson T. (2000). Pitfalls in forensic toxicology. Ann. Clin. Biochem..

[16] Linnet K. (1987). Two stage transformation systems for normalization of reference distributions evaluated. Clin. Chem..

[17] Grainger J., Huang W., Patterson D.G., Turner W.E., Pirkle J., Caudill S.P., Wang R.Y., Needham L.L., Sampson E.J. (2006). Reference range levels of polycyclic aromatic hydrocarbons in the U.S. population by measurement of urinary monohydroxy metabolites. Environ. Res..

[18] Seto H., Ohkubo T., Kanoh T., Koike M., Nakamura K., Kawahara Y. (1993). Determination of polycyclic aromatic hydrocarbons in the lung. Arch. Environ. Contam. Toxicol..

[19] Beach J.B., Pellizzari E., Keever J.T., Ellis L. (2000). Determination of benzo[a]pyrene and other polycyclic aromatic hydrocarbons (PAHs) at trace levels in human tissues. J. Anal. Toxicol..

[20] Gitipour S., Firouzbakht S., Mirzaee E., Alimohammadi M. (2014). Assessment of soil screening levels due to ingestion and dermal absorption of chrysene and benzo[k]fluoranthene and appropriate remediation method for Dorson Abad. Environ. Monit. Assess..

[21] Spink D.C., Wu S.J., Spink B.C., Hussain M.M., Vakharia D.D., Pentecost B.T., Kaminsky L.S. (2008). Induction of CYP1A1 and CYP1B1 by benzo(k)fluoranthene and benzo(a)pyrene in T-47D human breast cancer cells: Roles of PAH interactions and PAH metabolites. Toxicol. Appl. Pharmacol..

[22] Jung K.H., Liu B., Lovinsky-Desir S., Yan B., Camann D., Sjodin A., Li Z., Perera F., Kinney P., Chillrud S. (2014). Time trends of polycyclic aromatic hydrocarbon exposure in New York City from 2001 to 2012: Assessed by repeat air and urine samples. Environ. Res..

[23] Merlo F., Bolognesi C., Peluso M., Valerio F., Abbondandolo A., Puntoni R. (1997). Airborne levels of polycyclic aromatic hydrocarbons: ^32^P-postlabeling DNA adducts and micronuclei in white blood cells from traffic police workers and urban residents. J. Environ. Pathol. Toxicol. Oncol..

[24] Qin Y.Y., Leung C.K., Lin C.K., Leung A.O., Wang H.S., Giesy J.P., Wong M.H. (2011). Halogenated POPs and PAHs in blood plasma of Hong Kong residents. Environ. Sci. Technol..

[25] Ford J.G., Li Y., O’Sullivan M.M., Demopoulos R., Garte S., Taioli E., Brandt-Rauf P.W. (2000). Glutathione S-transferase M1 polymorphism and lung cancer risk in African-Americans. Carcinogenesis.

[26] O’Keefe S.J., Chung D., Mahmoud N., Sepulveda A.R., Manafe M., Arch J., Adada H., van der Merwe T. (2007). Why do African Americans get more colon cancer than Native Africans?. J. Nutr..

[27] Tang D., Kryvenko O.N., Wang Y., Jankowski M., Trudeau S., Rundle A., Rybicki B.A. (2013). Elevated polycyclic aromatic hydrocarbon-DNA adducts in benign prostate and risk of prostate cancer in African Americans. Carcinogenesis.

[28] Steck S.E., Butler L.M., Keku T., Antwi S., Galanko J., Sandler R.S., Hu J.J. (2014). Nucleotide excision repair gene polymorphisms, meat intake and colon cancer risk. Mutat. Res. Fundam. Mol. Mech. Mutagen..

[29] Tsang H.L., Wu S., Leung C.K., Tao S., Wong M.H. (2011). Body burden of POPs of Hong Kong residents, based on human milk, maternal and cord serum. Environ. Int..

[30] Wang S., Chanock S., Tang D., Li Z., Edwards S., Jedrychowski W., Perera F.P. (2010). Effect of gene-environment Interactions on mental development in African American, Dominican, and Caucasian mothers and newborns. Ann. Hum. Genet..

[31] Neal M.S., Zhu J., Foster W.G. (2008). Quantification of benzo[a]pyrene and other PAHs in the serum and follicular fluid of smokers *versus* non-smokers. Reprod. Toxicol..

[32] Pleil J.D., Stiegel M.A., Sobus J.R., Tabucchi S., Ghio A.J., Madden M.C. (2010). Cumulative exposure assessment for trace-level polycyclic aromatic hydrocarbons (PAHs) using human blood and plasma analysis. J. Chromatogr. B Analyt. Technol. Biomed. Life Sci..

[33] Al-Daghri N.M., Alokail M.S., Abd-Alrahman S.H., Draz H.M. (2014). Polycyclic aromatic hydrocarbon distribution in serum of Saudi children using HPLC-FLD: Marker elevations in children with asthma. Environ. Sci. Pollut. Res. Int..

[34] Hussar E., Richards S., Lin Z.Q., Dixon R.P., Johnson K.A. (2012). Human health risk assessment of 16 priority polycyclic aromatic hydrocarbons in soils of Chattanooga, Tennessee, USA. Water Air Soil Pollut..

[35] Kim J.H., Yamaguchi K., Lee S.H., Tithof P.K., Sayler G.S., Yoon J.H., Baek S.J. (2005). Evaluation of polycyclic aromatic hydrocarbons in the activation of early growth response-1 and peroxisome proliferator activated receptors. Toxicol. Sci..

[36] Williams S.D., Ladd D.E., Farmer J.J. (2006). Fate and Transport of Petroleum Hydrocarbons in Soil and Ground Water at Big South Fork National River and Recreation Area, Tennessee and Kentucky, 2002–2003.

[37] Wang N., Ingersoll C.G., Kunz J.L., Brumbaugh W.G., Kane C.M., Evans R.B., Alexander S., Walker C., Bakaletz S. (2013). Toxicity of sediments potentially contaminated by coal mining and natural gas extraction to unionid mussels and commonly tested benthic invertebrates. Environ. Toxicol. Chem..

[38] Ruhl L., Vengosh A., Dwyer G.S., Hsu-Kim H., Deonarine A., Bergin M., Kravchenko J. (2009). Survey of the potential environmental and health impacts in the immediate aftermath of the coal ash spill in Kingston, Tennessee. Environ. Sci. Technol..

[39] Fu Z., Shrubsole M.J., Li G., Smalley W.E., Hein D.W., Cai Q., Ness R.M., Zheng W. (2013). Interaction of cigarette smoking and carcinogen-metabolizing polymorphisms in the risk of colorectal polyps. Carcinogenesis.

[40] Fu Z., Shrubsole M.J., Smalley W.E., Wu H., Chen Z., Shyr Y., Ness R.M., Zheng W. (2011). Association of meat intake and meat-derived mutagen exposure with the risk of colorectal polyps by histologic type. Cancer Prev. Res..

